# Estimating the chance of success in IVF treatment using a ranking algorithm

**DOI:** 10.1007/s11517-015-1299-2

**Published:** 2015-04-17

**Authors:** H. Altay Güvenir, Gizem Misirli, Serdar Dilbaz, Ozlem Ozdegirmenci, Berfu Demir, Berna Dilbaz

**Affiliations:** Computer Engineering Department, Bilkent University, 06800 Ankara, Turkey; Department of Obstetrics and Gynecology, Duzce University School of Medicine, 81260 Duzce, Turkey; Zekai Tahir Burak Women’s Health Education and Research Hospital, 06230 Ankara, Turkey; Etlik Zubeyde Hanim Women’s Health, Teaching and Research Hospital, 06010 Ankara, Turkey

**Keywords:** Estimation of success, Ranking, Classification, In vitro fertilization, Clinical decision support system

## Abstract

In medicine, estimating the chance of success for treatment is important in deciding whether to begin the treatment or not. This paper focuses on the domain of in vitro fertilization (IVF), where estimating the outcome of a treatment is very crucial in the decision to proceed with treatment for both the clinicians and the infertile couples. IVF treatment is a stressful and costly process. It is very stressful for couples who want to have a baby. If an initial evaluation indicates a low pregnancy rate, decision of the couple may change not to start the IVF treatment. The aim of this study is twofold, firstly, to develop a technique that can be used to estimate the chance of success for a couple who wants to have a baby and secondly, to determine the attributes and their particular values affecting the outcome in IVF treatment. We propose a new technique, called success estimation using a ranking algorithm (SERA), for estimating the success of a treatment using a ranking-based algorithm. The particular ranking algorithm used here is RIMARC. The performance of the new algorithm is compared with two well-known algorithms that assign class probabilities to query instances. The algorithms used in the comparison are Naïve Bayes Classifier and Random Forest. The comparison is done in terms of area under the ROC curve, accuracy and execution time, using tenfold stratified cross-validation. The results indicate that the proposed SERA algorithm has a potential to be used successfully to estimate the probability of success in medical treatment.

## Introduction

Assisted reproductive technologies (ART) give infertile couples a chance to have a baby. The first baby born using in vitro fertilization (IVF) was in 1978. Since 1978, different techniques, including intracytoplasmic sperm injection, pre-implantation genetic diagnosis, gamete and embryo cryopreservation, have been used as new treatment options for clinicians to achieve greater success.

Widespread use of the Internet provides information to infertile couples and raises their awareness of treatment options. Couples with infertility problems want to know their chances of having a baby when selecting the best treatment options that are based on their underlying pathology. Since the cost of IVF treatment per cycle is very high, estimating the chance of success rate per treatment cycle by using patients’ personal parameters constitutes a great advantage in the field of reproductive medicine.

Machine learning techniques can be used to analyze a clinical database, including patient characteristics, all available data for ovarian hyperstimulation and pregnancy outcome. The models learned by these techniques can be used to estimate the probability of success for an infertile couple.

In this paper, we propose a new technique, called SERA, for success estimation using ranking algorithms, which can be used for estimating the probability of success for a treatment cycle. The SERA algorithm can be used with any ranking algorithm that assigns each instance a score value to be used for ranking. We exemplify the proposed method using a dataset collected from IVF treatment records in a hospital. For a new patient couple, such a ranking method assigns a score to the couple and determines its rank among the training instances. Then, the chance of the treatment success for the new couple can be estimated as the ratio of successful training instances among those with similar score values.

The SERA algorithm, proposed in this study, is implemented using the RIMARC (ranking instances by maximizing the area under ROC curve) algorithm [11]. There are several reasons for choosing the RIMARC algorithm for SERA. Firstly, the model constructed by the RIMARC algorithm is a set of human interpretable rules that indicate the attributes and their particular values affecting the outcome. Therefore, the medical experts can validate the risk factors affecting the outcome in the IVF treatment. Another important characteristic of RIMARC is that it has no parameters that need to be tuned for the optimum performance. Therefore, there is no need for a parameter optimization step after the edition of new training data. Finally, the RIMARC algorithm is robust to missing feature values; in that, it does not require imputation of artificial values nor removal of cases or features with missing values; and it uses whatever data are available.

In IVF treatment, couples would like to know the chance of success considering their underlying pathology. An infertile couple would like to decide whether to go on with treatment considering the chance and the risks they are willing to take. We measure the success of such a chance estimation algorithm mainly in terms of AUC (area under ROC curve). We compared SERA with some well-known algorithms that assign class probabilities to query instances. The algorithms used in the comparison are Naïve Bayes Classifier [8] and Random Forest [2]. The comparison is done in terms of both AUC and accuracy, using tenfold stratified cross-validation. The results indicate that the proposed SERA algorithm is successful in estimating the probability of success in a medical treatment.

Another aim of this study is to determine the attributes and their particular values that affect the outcome of an IVF treatment. This is done by executing the RIMAC algorithm using the whole cohort as the training data. RIMARC learns a rule for each feature. The rules are in a form interpretable by experts in the domain. These results help the experts to validate the model constructed for predicting the outcome.

The remainder of the paper is organized as follows: Sect. 2 introduces the IVF dataset used in this study. In Sect. 3, the proposed SERA algorithm is described. The SERA algorithm is compared with four well-known classification algorithms. The results of the experiments and some of the rules learned by RIMARC are given in Sect. 4. These rules and related work are discussed in Sect. 5. Finally, Sect. 6 concludes with future research directions.

## Dataset

A dataset of 1456 patients has been compiled by the IVF Unit at Etlik Zubeyde Hanim Women’s Health, Teaching and Research Hospital, located in Ankara, Turkey. This study was approved by the Local Education Planning Committee of the hospital. For each patient, the dataset contains demographic and clinical parameters, as independent features. We formed the dataset for the experiments by taking only the features that are potentially relevant for the algorithms. The dataset has one dependent feature, called *Result*, that has the value *P* (for success) if the woman had a clinical pregnancy which is defined as the detection of fetal heart beat on the ultrasound examination, and the value *N* (for failure) if the patient had only a chemical pregnancy or no pregnancy, at all. The number of *P* labeled cases is 423, and the *N* labeled cases is 1033. Therefore, the default accuracy is 0.709, predicting the class label of all query instances as *N*.

The IVF dataset contains only the clinical features that are known before making a decision to proceed with the IVF treatment. The dataset contains 64 independent features; 52 of them are related to the female, and 12 are related to the male. The independent features included in the IVF dataset are summarized in Table 1. Among the independent features, 43 of them take on categorical values and 21 of them are numerical. Categorical features are indicated with a (C), binary (categorical feature with only two values) ones with a (B) and numerical ones with a (N). Features that take on only binary values, such as Yes/No or True/False, are treated as categorical. About 13.5 % of the feature values in the dataset are missing.

**Table 1:** Features in IVF dataset (N: numeric, C: Categorical, B: Binary)

Variables related to female		Variables related to male
Female_Age (N)	Laparoscopy (C)	Male_Factor (B)
Female_Blood_Type (C)	Hysteroscopy (C)	Male_Age (N)
Height (N)	Laparoscopic_Surgery (C)	Male_Blood_Type (C)
Weight (N)	Hysteroscopic_Surgery (C)	Male_Genital_Surgery (C)
BMI* (N)	Abdominal_Surgery (C)	Semen_Analysis_Category (C)
Tubal_Factor (B)	Abdominal_Surgery_Category (C)	Male_FSH (N)
Age_Related_Infertility (B)	Gynecologic_Surgery (C)	Sperm_Count (N)
Ovulatory_Dysfunction (B)	Ovarian_Surgery (C)	Sperm_Motility(N)
Unexplained_Infertility (B)	Tubal_Surgery (C)	Total_Progressive_Sperm_Count (N)
Severe_Pelvic_Adhesion (B)	Uterine_Surgery (C)	Sperm_Morphology (N)
Endometriosis (B)	Duration_Infertility (N)	Testicular_Biopsy (C)
Cycle_No. (N)	PCOS* (B)	TESE*_Outcome (C)
Baseline_FSH* (N)	HSG*_Cavity (C)	Male_Karyotype (C)
Baseline_LH* (N)	HSG*_Tubes* (C)	
Baseline_E2* (N)	Hydrosalpinx (C)	
G* (N)	Office_Hysteroscopy(C)	
A* (N)	Office_Hysteroscopic_Incision (B)	
Y* (N)	Office_Hysteroscopic_Procedure (C)	
DM* (C)	Total_Antral_Follicle_Count (N)	
HT* (B)	Right_Ovarian_Antral_Follicle_Count (N)	
Thyroid_Disease (C)	Left_Ovarian_Antral_Follicle_Count (N)	
Anemia (B)	Hyperprolactinemia (B)	
Laparotomy (C)	Hepatitis (C)	
Cyst_Aspiration (B)	Endometrioma_Surgery (C)	
Embryocryo (B)	Localization_Myoma_Uteri (C)	

* *BMI* body mass index, *FSH* follicle-stimulating hormone, *LH* luteinizing hormone, *E2* estradiol, *G* gravida, *A* abortus, *Y* living children, *DM* diabetes mellitus, *HT* hypertension, *PCOS* polycystic ovary syndrome, *HSG* hysterosalpingography, *TESE* testicular sperm extraction

## Proposed estimation algorithm

The proposed success estimation algorithm in this study, SERA, uses RIMARC as its ranking algorithm. Therefore, we first sketch the RIMARC algorithm with an example of computing the score for a patient couple, and then the SERA algorithm is described.

### The RIMARC algorithm

RIMARC is a supervised algorithm that learns a scoring function to rank instances [11]. It does not make any assumptions about the data and has no parameters to tune for optimizing the performance. The RIMARC algorithm aims to maximize the AUC value, since the area under the ROC curve (AUC) has become a widely accepted performance evaluation metric in evaluating the quality of ranking. It learns a ranking function which is a linear combination of nonlinear score functions constructed separately for each feature. Each of these nonlinear score functions aims to maximize the AUC by considering only the corresponding feature in ranking. It has been shown that, for a single categorical feature, it is possible to derive a scoring function that achieves the maximum possible AUC. Therefore, the RIMARC algorithm first discretizes all continuous features into categorical ones, in a way that optimizes the AUC, by using the MAD2C algorithm proposed by Kurtcephe and Güvenir [18].

A categorical feature *f* has a finite set of values. Let *V*_*f*_ = {*v*_1_, *v*_2_, …, *v*_k_} be the set of values for a given categorical feature *f*. Consider a dataset that includes only this feature and a class value for each instance. That is, an instance is represented by two values: *f* value and class label. A scoring function *s*_*f*_() can be defined to rank the elements of *V*_*f*_. According to this scoring function, $v_{\text{i}} \,{ \preccurlyeq }\, v_{\text{j}}$ if and only if $s_{f} \left( {v_{\text{i}} } \right) \le s_{f} \left( {v_{\text{j}} } \right)$. Note that the problem of ranking the instances in a dataset is reduced to the problem of ranking the values of a feature. Güvenir and Kurtcephe showed that a scoring function has to satisfy the following condition in order to achieve the maximum AUC [11]:

1 $$s_{f} \left( {v_{\text{i}} } \right) \le s_{f} \left( {v_{\text{j}} } \right)\,\,{\text{iff}}\,\,\frac{{P_{\text{i}} }}{{N_{\text{i}} }} < \frac{{P_{\text{j}} }}{{N_{\text{j}} }}$$

Here *P*_i_ is the number of positive (*P* labeled) instances and *N*_i_ is the number of negative (*N* labeled) instances, for the value *v*_i_ of feature *f*. Note that any scoring function that satisfies this condition will result in the maximum possible AUC in ranking the dataset with a single feature. It is important to note that, for some values of *i*, *N*_i_ may be 0. In such cases, the ranking function will have an undefined value. In order to overcome this problem, the RIMARC algorithm defines the ranking function as follows:

2 $$s_{f} \left( {v_{\text{i}} } \right) = \frac{{P_{\text{i}} }}{{P_{\text{i}} + N_{\text{i}} }}$$

This newly defined scoring function satisfies the condition in Eq. (1) and furthermore is interpretable by medical doctors, since it is simply the probability of the *p* label among all instances with value *v*_i_. This probability value is easily interpretable by humans. The instances of the dataset, which has a single categorical feature *f*, are sorted by the scoring function *s*_*f*_(), and the AUC is computed. The AUC obtained by this scoring function is guaranteed to be between 0.5 and 1.0 [11]. If the feature *f* is irrelevant, the AUC will be 0.5. On the other hand, if the single feature *f* is sufficient to predict the class label, all positive and negative instances will be separated by the scoring function *s*_*f*_(), and the AUC will be 1.0. The RIMARC algorithm uses the AUC value to measure the weight (relevancy) of the feature *f* as:

3 $$w_{f} = 2\left( {{\text{AUC}}_{f} {-}0.5} \right) ,$$

where AUC_*f*_ is the AUC obtained for feature *f*. Therefore, the weight of a feature will be in the range of [0, 1]. The RIMARC algorithm computes the weight of each feature by setting up a sub-dataset, which is composed of only that feature and the class.

As an example, suppose that the AUC computed for the feature *f* is 1. This means perfect ordering, and this is the maximum value that AUC can have. That is, all instances in the training set can be ranked by using only the values of feature *f*. Therefore, we expect that query instances can be ranked correctly among the training set by using only feature *f*.

The rule model learned by the RIMARC algorithm is used to compute the score for a given query patient *q* as:

4 $$\begin{array}{*{20}l} {{\text{score}}\left( q \right) = \frac{{\mathop \sum \nolimits_{f} w_{f}^{q} . s_{f} \left( q \right)}}{{\mathop \sum \nolimits_{f} w_{f}^{q} }}} \hfill \\ {w_{f}^{q} = \left\{ {\begin{array}{*{20}l} {w_{f} } \hfill & {q_{f} {\text{is known}}} \hfill \\ 0 \hfill & {q_{f} {\text{is missing}}} \hfill \\ \end{array} } \right.} \hfill \\ \end{array}$$

Here *w*_*f*_ represents the weight of the feature *f*, and *s*_*f*_(*q*) represents the score associated with the value of feature *f* for patient couple *q*, queried. The RIMARC algorithm is robust to missing feature values. The features whose values in query *q* are missing are simply ignored when computing the score for that query. For example, consider a 25-year-old female, whose BMI is 25.7, she does not have age-related infertility, the semen analysis category for her partner is asthenospermia, and the values of all other features are missing. Then the score of the treatment for this couple can be computed as shown in Table 2.

**Table 2:** An example of computing score using RIMARC

Feature	Feature weight *w*_*f*_	Feature value	Score value *s*_*f*_(*q*)	Weighted score *w*_*f*_ *·s*_*f*_(*q*)
Female_Age	0.1753	25	0.2375	0.0416
BMI	0.1443	25.7	0.2169	0.0313
Semen_Analysis_Category	0.1407	Astheno	0.3571	0.0503
Age_Related_Infertility	0.1178	No	0.2245	0.0264
**Sum**	**0.5781**			**0.1496**
Score(*q*) = 0.1496/0.5781 = **0.2587**				

Bold values indicate the results

In summary, the RIMARC ranking algorithm does not have parameters that have to be tuned after the addition of new records about existing or new patients. Further, the ranking knowledge constructed by RIMARC includes information about the importance of features as weights and effects of particular values or ranges in the success of the treatment in terms of scores. This form of knowledge can be analyzed and verified by the domain experts.

Another important characteristic of the REMARC algorithm is its robustness to missing feature values. Since it processes each feature individually, missing feature values are simply ignored when processing the corresponding feature. Therefore, instead of ignoring a complete patient record with missing feature values, or imputing them with artificial values, it uses all data available about a patient.

### The SERA algorithm

The SERA algorithm is designed to estimate the chance of a treatment as the probability of success of the treatment for a patient couple. It uses the score assigned to the couple by a ranging algorithm to determine the similar past cases. Although it can be any ranking algorithm that assigns score values for the instances, it uses the RIMARC algorithm for the reasons given above. The ranking score value is used to locate the query patient among the training cases. However, what we need is the chance of success for a new infertile couple. On the other hand, semantically, the word “chance” refers to the probability. In order to calculate the chance of success of IVF treatment for a query patient *q*, we select the first *k* (e.g., *k* = 100) past (training) patients whose ranking scores are closest to score(*q*), the score computed by the query couple. If the number of successful cases among these *k* similar training cases is *P*_count_, then the chance of success for *q* is reported as

5 $${\text{chance}}\left( q \right) = \frac{{P_{\text{count}} }}{k}$$

That is, chance(*q*) represents the probability of success considering the most similar *k* past cases in terms of the score values. An alternative approach, to determine the neighbors, is to fix the radius *r*, and select all training instances *p*, such that $\left| {{\text{score}}\left( q \right) - {\text{score}}\left( p \right)} \right| < r$, as the neighbors of *q*.

**Fig. 1:**
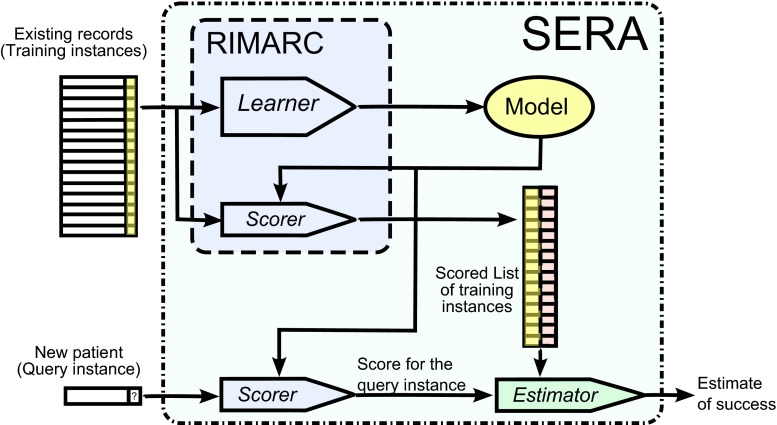
Block diagram of the SERA algorithm

It should be noted that the chance(*q*) is the probability among the past instances with similar score values. In reality, one would be interested in patient similarity in terms of metrics such as Euclidean distance. Similar instances have similar score values, whereas instances with similar score values may look different. This might appear to be a limitation. However, using a successful scoring function, it leads to efficient searches for similar cases. The block diagram of the SERA algorithm is given in Fig. 1.

The SERA algorithm can also be used for binary classification, where the class labels are *P* and *N*. As shown in Eq. (6), the class label of a query instance *q* can be predicted as *P* if the chance(*q*) is higher than 50 %.

6 $${\text{prediction(}}q{\text{)}} = \left\{ {\begin{array}{*{20}l} P \hfill & {\quad {\text{chance}}(q) > 0.5} \hfill \\ N \hfill & {\quad {\text{otherwise}}} \hfill \\ \end{array} } \right.$$

## Results

In this section, we present a comparison of the SERA algorithm with some well-known classification algorithms that assign class probabilities to query instances. The algorithms used in the comparison are Naïve Bayes Classifier [8] and Random Forest [2]. Despite their simplicity, Naïve Bayes Classifiers have worked well in many complex real-world situations. Further, they are suited when the dimensionality of the inputs is high. The choice of Random Forest is due to the fact that they cope with the problem of overfitting the training data by averaging multiple deep decision trees, trained on different parts of the same training set, with the goal of reducing the variance [13].

The comparison is performed in terms of area under the ROC curve and accuracy, using tenfold stratified cross-validation, over 200 random datasets obtained by shuffling the original dataset. Tests for these algorithms are performed using the Weka toolbox, which is a collection of machine learning algorithms, implemented in the Java language [12]. The Naïve Bayes Classifier and Random Forest algorithms are implemented as java classes as Naive Bayes and Random Forest. These classes replace all missing values for nominal and numeric attributes in a dataset with the modes and means from the training data, respectively. In our experiments, all parameters for the algorithms are set to their default values. Although, in general, classification algorithms are badly affected from the curse of dimensionality, we did not apply any technique to reduce the number of features, since one of the aims of this study is to determine the attributes and their particular values that affect the outcome of an IVF treatment.

The SERA algorithm is also implemented in the Java language. The number of nearest neighbors considered by SERA is set to 100 (default). Table 3 displays the results of the experiments^1^ conducted using the IVF dataset. As seen from the table, SERA outperforms the other classifiers in terms of both AUC and accuracy. Also SERA is the second fasted algorithm in terms of the execution time.

**Table 3:** Results of AUC, accuracy and execution time on the IVF dataset

Algorithm	AUC	Area under ROC curve			Accuracy	Execution time (s)
		SE	95 % Confidence interval			
			Lower bound	Upper bound		
SERA	0.833 (±0.003)	0.012	0.809 (±0.003)	0.857 (±0.004)	0.844 (±0.004)	1.4 (±0.2)
NBC	0.794 (±0.002)	0.014	0.767 (±0.002)	0.822 (±0.002)	0.783 (±0.002)	0.8 (±0.1)
Random forest	0.769 (±0.009)	0.014	0.741 (±0.010)	0.797 (±0.008)	0.792 (±0.008)	2.0 (±0.1)

The values are mean (±SD) over 200 runs with random shuffling of the dataset (*p* < 0.0005 for all algorithms)

**Fig. 2:**
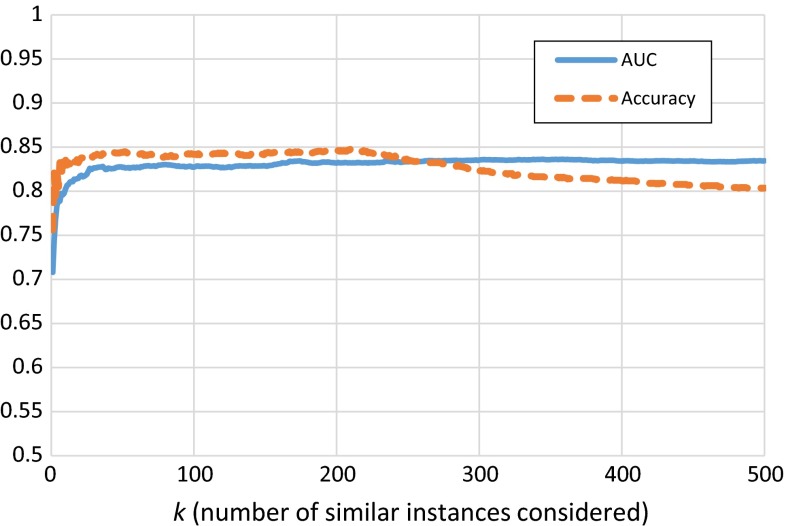
Effect of *k* on AUC and accuracy

We further experimented with the choice of *k* (number of similar instances considered) on the AUC and accuracy with the IVF dataset. Both AUC and accuracy remain almost constant for values *k* > 40. The accuracy slightly drops when *k* > 230. The graph is shown in Fig. 2.

**Fig. 3:**
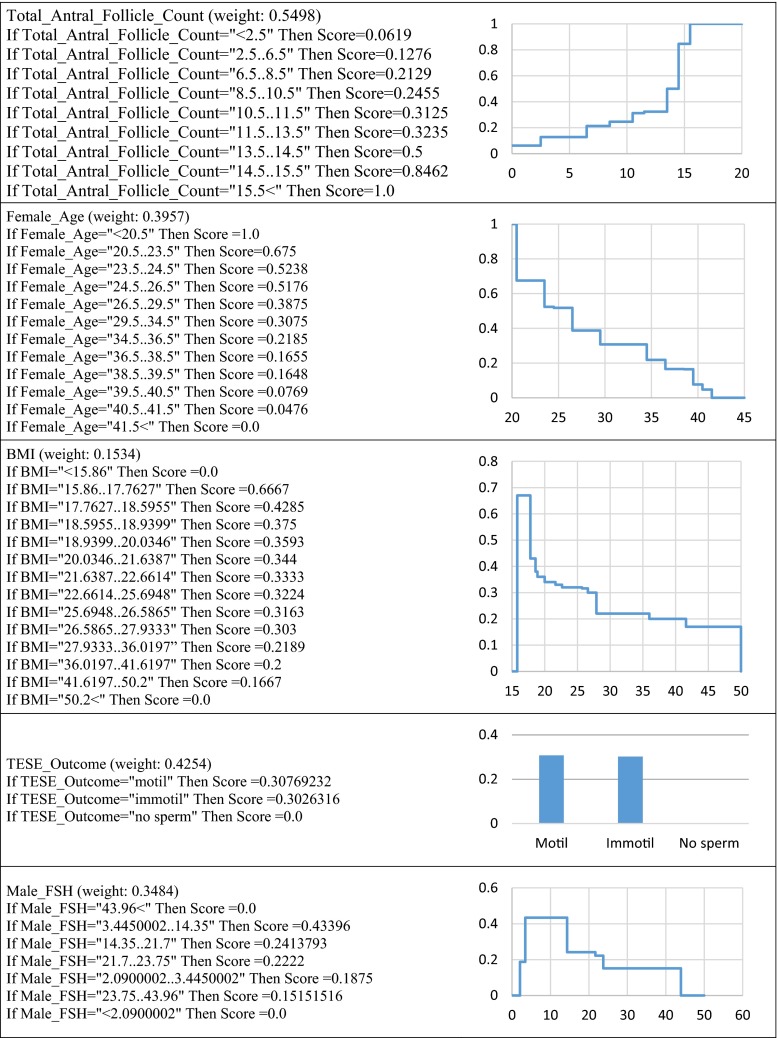
Some rules learned by RIMARC

Another aim of this study is to determine the attributes and their particular values that affect the outcome of an IVF treatment. This is done by executing the RIMAC algorithm using the whole cohort as the training data. RIMARC learns a rule for each feature. The rules are in form interpretable by experts in the domain. These rules help the experts to validate the model constructed for predicting the outcome. The feature weights learned by RIMARC, using the whole cohort as the training set, are shown in Table 4. For continuous attributes, the threshold values over or below which the chances of success change drastically are very valuable. The RIMARC algorithm determines these threshold values by discretizing the continuous attributes using the MAD2C algorithm [18]. Some of the rules learned for some of the features, using the whole cohort as the training set to RIMARC, are shown in Fig. 3.

**Table 4:** Feature weights learned by RIMARC

Feature	Weight	Feature	Weight
Laparoscopic_Surgery	0.6455	Laparotomy	0.0909
Total_Antral_Follicle_Count	0.5498	Male_Karyotype	0.0834
Right_Ovarian_Antral_Follicle_Count	0.5163	HSG_Tubes	0.0783
Left_Ovarian_Antral_Follicle_Count	0.4934	Myoma_Uteri	0.0737
Hysteroscopic_Surgery	0.457	Uterine_Surgery	0.0711
TESE_Outcome	0.4254	Sperm_Morphology	0.0708
Female_Age	0.3957	Abdominal_Surgery	0.053
Male_FSH	0.3484	Cycle_No	0.0508
Male_Blood_Type	0.3118	Tubal_Factor	0.0496
Male_Age	0.2777	Cyst_Aspiration	0.0384
Baseline_FSH	0.2764	Ovarian_Surgery	0.0354
PCOS	0.2258	Male_Factor	0.0332
Total_Progressive_Sperm_Count	0.2151	Endometrioma_Surgery	0.0276
Sperm_Count	0.2098	Abdominal_Surgery_Category	0.0274
Localization_Myoma_Uteri	0.2021	Thyroid_Disease	0.0273
Age_Related_Infertility	0.1959	Testicular_Biopsy	0.0256
Ovulatory_Dysfunction	0.1791	Laparoscopy	0.0231
Gynecologic_Surgery	0.1779	Hysteroscopy	0.0197
Semen_Analysis_Category	0.1777	DM	0.0141
Unexplained_Infertility	0.1775	Tubal_Surgery	0.0125
Duration_Infertility	0.1567	HT	0.0122
BMI	0.1534	Y	0.012
Height	0.1339	Endometriosis	0.0118
Weight	0.1333	Embryocryo	0.0117
Female_Blood_Type	0.127	Hydrosalpinx	0.0104
Office_Hysteroscopic_Procedure	0.1245	G	0.0101
Office_Hysteroscopy	0.1238	Office_Hysteroscopic_Incision	0.01
Baseline_LH	0.1196	A	0.0093
HSG_Cavity	0.1048	Hyperprolactinemia	0.0085
Male_Genital_Surgery	0.1039	Hepatitis	0.0079
Sperm_Motility	0.096	Severe_Pelvic_Adhesion	0.0047
Baseline _E2	0.095	Anemia	0.0004

Reproductive aging occurs as a consequence of the decrease in the quantity and quality of the ovarian follicles [34]. Approximately 1000 follicles are depleted per month during the reproductive period, and this depletion increases significantly after the age of 35 [9]. The antral follicle count is a reliable diagnostic test for the evaluation of ovarian reserve. Ovarian reserve tests reflect the quantitative aspect of the ovarian reserve status. These tests are mainly used to predict the response of the ovarian hyperstimulation. However, prediction of the success of IVF treatment is mainly based on the quality of the oocytes. Recently, a meta-analysis by Broer et al. [3] assessed the additional value of the ovarian reserve test in predicting IVF success. According to the results of the RIMARC, female age and total antral follicle count are significant predictors of IVF success. Clinical pregnancy rates significantly decrease after the age of 34.5 and when the total antral follicle count falls below 10.5.

The impact of obesity on the outcome of infertility treatment is a contentious issue. While some studies associate obesity with the need for higher doses of gonadotropins, increased cycle cancellation rates, fewer oocyte yield and higher miscarriage rates [10, 24, 32], other studies have been unable to find any negative effect of obesity on IVF outcome [7, 20]. In experiments with RIMARC, an important decrease in the clinical pregnancy rate was observed when the BMI was higher than 27.9.

## Discussion

Infertility is defined as the failure to conceive after 12 months or more of regular unprotected intercourse [29]. In 2010, among women 20–44 years of age who were exposed to the risk of pregnancy, 1.9 % were unable to attain a live birth (primary infertility) [25]. Of the women who had had at least one live birth and were exposed to the risk of pregnancy, 10.5 % were unable to have another child (secondary infertility). The major causes of infertility are as follows: ovulatory dysfunction (15 %), tubal and peritoneal pathology (35 %), male infertility (35 %), unexplained infertility (10 %) and unusual reasons (5 %) [4, 15].

IVF involves a sequence of coordinated procedures that begin with controlled ovarian hyperstimulation (COH), followed by retrieval of oocytes from the ovaries under the guidance of transvaginal ultrasonography, fertilization of the oocytes and spermatozoa at the embryology laboratory, and finally, embryo transfer into the uterine cavity.

Controlled ovarian hyperstimulation is used to induce the growth of multiple follicles. Numerous treatment regimens have been described; the most preferred agents are gonadotropins in combination with a gonadotropin-releasing hormone (GnRH) agonists or antagonists. GnRH agonists or antagonists are mainly used to prevent a premature LH surge. COH protocols are described according to the use of oral contraceptives, timing and duration of GnRH agonists such as the long, short, micro-dose flare up, and stop protocol. The selection of the COH protocols in clinical practice is based mainly on the patient’s age and ovarian reserve (poor or hyperresponder).

FSH-containing gonadotropins are used for ovarian stimulation. Human menopausal gonadotropins are extracted from the urine of postmenopausal women. Highly purified urinary FSH preparations with no contaminating urinary proteins are produced. Advanced technology is used to produce recombinant gonadotropins that are free of contamination of proteins and viruses. All gonadotropins are orally inactive [1]. Ovarian reserve and body mass index are important parameters in the determination of the daily gonadotropin dosage.

Oocyte retrieval is generally performed approximately 34–36 h after hCG administration. The standard technique of oocyte retrieval is performed under the guidance of the transvaginal ultrasonography with intravenous sedation anesthesia.

To as close as possible to the time of the oocyte retrieval, a semen sample is obtained by masturbation. If a patient has no sperm in the ejaculate (azoospermia), a variety of surgical approaches is used for sperm extraction. Microscopic testicular sperm extraction is the most complicated procedure among them.

In the IVF procedure, an oocyte is incubated with 100,000–200,000 motile spermatozoa in vitro. If the selected spermatozoa is injected into the ooplasm using an injection pipette under a microscope, this procedure is called intracytoplasmic sperm injection.

The main important factors affecting the success rate of IVF are woman’s age and the cause of infertility. Other prognostic factors are as follows: hydrosalpinx, uterine myomas, smoking and obesity.

Clinical pregnancy is defined as the presence of an intrauterine gestational sac with fetal cardiac activity as confirmed by transvaginal ultrasonography. Chemical pregnancy is accepted as low level of βhCG that is not confirmed through visualization of the gestational sac.

We showed that machine learning systems could help to determine the relative weights of the risk factors and the relative effects of the particular values of these risk factors. Most importantly, machine learning systems could predict the personalized chance of the outcome related to these factors to help decide whether to start the complex IVF treatment procedure.

IVF treatment is a long, complex and costly process. It is very stressful for couples who want to have a baby. Although there many machine learning applications for clinical decision support systems in the literature [6, 19, 22, 27], systems related to obstetrics is limited [28]. The literature shows that in early studies, case-based reasoning systems and neural networks have been constructed to predict the outcome of IVF [16, 17]. Subsequently, decision tree models are constructed to predict the outcome of IVF treatment [33, 34]. The most recent studies on IVF propose Naive Bayes, Bayesian Classification and Support Vector Machines to increase the chance of having a baby after IVF treatment. Uyar et al. [35] studied for implantation prediction on IVF embryos using Naive Bayes classification. In another study, the embryo implantation prediction is defined. In this study, embryo-based prediction is identified in order to predict the outcome of IVF treatment and an SVM-based learning system is used [37]. In addition, there is a study related to predicting implantation potentials of IVF embryos [36]. Predicting the IVF outcome is a considerably challenging process, so much research aims to address this problem [5, 26].

The area under the ROC curve (AUC) is a widely accepted performance measure for evaluating the quality of ranking [21]. It has become a popular performance measure in the machine learning community after it was discovered that accuracy is often a poor metric to evaluate classifier performance [13, 14, 23, 30, 31].

## Conclusions

In vitro fertilization is a common infertility treatment method in which female oocytes are inseminated by sperm under laboratory conditions. Given a new candidate for IVF, the first important consideration is whether to apply the IVF treatment or not. The decision is made mainly by the clinician and the couple. Since the IVF treatment involves an application of several hormones and medicines to both female and male patients, it is a difficult and stressful process. If the chances of success are low, the couple may choose not to start the treatment. However, as in many areas of medicine it is not possible to construct a mathematical model that, given the values of relevant parameters for a couple, returns the outcome of the IVF treatment.

In this paper, we showed that it is possible to learn a model, from a set of past cases of IVF treatment, which can estimate the outcome of the treatment for a given couple. We tested three such score-based ranking algorithms, namely SERA, Naïve Bayesian Classifier and Random Forest. These supervised machine learning algorithms applied to a dataset of cases learn a model that can be used to estimate the likelihood of success. We applied these algorithms to a dataset of cases, where each case, called a cycle, represents the values of parameters that are measured before applying the IVF treatment, along with the outcome of the treatment.

The RIMARC algorithm, used by SERA, has three important characteristics for medical applications. Firstly, it learns rules about the data, which can be further analyzed by medical practitioners. Secondly, it does not have parameters that need to be optimized after the addition of new patient records. Finally, it is robust to missing feature values, which is common in medical datasets. Further, the results of our experiments showed that the SERA algorithm outperformed the others in terms of both AUC and accuracy.

Further, the RIMARC algorithm calculates feature weights and creates rules that are in a human-readable form and easy for clinicians to interpret. This characteristic of RIMARC enables clinicians to validate the model constructed.

As a future work, we plan to collect similar datasets from other IVF clinics and apply the SERA algorithm. We will investigate whether the models agree with the one constructed in this study. If the models differ to a large extent, then the possible difference in the patient profile should be investigated. Further, we plan to apply the SERA algorithm to datasets from other disciplines of medicine.

## Footnotes

1 Experimented are performed on a PC with 64-bit Windows 7 OS, 3 GHz, Core2Duo CPU and 4 GB RAM.
